# Novel high dose rate lip brachytherapy technique to improve dose homogeneity and reduce toxicity by customized mold

**DOI:** 10.1186/s13014-014-0271-x

**Published:** 2014-12-23

**Authors:** Jon Feldman, Limor Appelbaum, Mordechay Sela, Ninel Voskoboinik, Sarit Kadouri, Jeffrey Weinberger, Itzhak Orion, Amichay Meirovitz

**Affiliations:** Oncology Department, Radiation Therapy Unit, Hadassah – Heberew University Medical Center, PO Box 12000, Jerusalem, (9112001) Israel; Department of Nuclear Engineering, Ben Gurion University of the Negev, Beer Sheva, (84105) Israel; Maxillofacial Rehabilitation department, Hadassah – Heberew University Medical Center, PO Box 12000, Jerusalem, (9112001) Israel; Department of Otolaryngology, Hadassah – Heberew University Medical Center, (PO Box 12000), Jerusalem, (9112001) Israel

**Keywords:** Brachytherapy, Lip, SCC, HDR, Customized mold technique

## Abstract

**Purpose/Objectives:**

The purpose of this study is to describe a novel brachytherapy technique for lip Squamous Cell Carcinoma, utilizing a customized mold with embedded brachytherapy sleeves, which separates the lip from the mandible, and improves dose homogeneity.

**Materials and methods:**

Seven patients with T2 lip cancer treated with a “sandwich” technique of High Dose Rate (HDR) brachytherapy to the lip, consisting of interstitial catheters and a customized mold with embedded catheters, were reviewed for dosimetry and outcome using 3D planning. Dosimetric comparison was made between the “sandwich” technique to “classic” – interstitial catheters only plan. We compared dose volume histograms for Clinical Tumor Volume (CTV), normal tissue “hot spots” and mandible dose. We are reporting according to the ICRU 58 and calculated the Conformal Index (COIN) to show the advantage of our technique.

**Results:**

The seven patients (ages 36–81 years, male) had median follow-up of 47 months. Four patients received Brachytherapy and External Beam Radiation Therapy, 3 patients received brachytherapy alone. All achieved local control, with excellent esthetic and functional results. All patients are disease free.

The Customized Mold Sandwich technique (CMS) reduced the high dose region receiving 150% (V150) by an average of 20% (range 1–47%), The low dose region (les then 90% of the prescribed dose) improved by 73% in average by using the CMS technique. The COIN value for the CMS was in average 0.92 as opposed to 0.88 for the interstitial catheter only. All differences (excluding the low dose region) were statistically significant.

**Conclusion:**

The CMS technique significantly reduces the high dose volume and increases treatment homogeneity. This may reduce the potential toxicity to the lip and adjacent mandible, and results in excellent tumor control, cosmetic and functionality.

## Introduction

Lip and oral cavity cancer is the 15th most common cancer worldwide, and the 15th most common cancer in Europe with more than 300,000 and around 61,400 new cases diagnosed in 2012 respectively (in both cases 2% of the total) [1]. The incidence in Europe of oral cavity and pharynx cancer in 2012 was 99.6 per 100,000 [2]. In some areas of the world, especially those with a lot of sunshine, the most common sub-site for cancer of the oral cavity is the lip [3]. The most frequent histologic type of lip cancer is Squamous Cell Carcinoma. This is due to the increased exposure to UV radiation in fair-skinned individuals living in regions exposed to sun.

Due to its location, lip cancer is usually detected at an early stage and single-modality therapy will usually suffice. Surgery or radiotherapy can be utilized, with similar local control and overall survival results [4-6].

The extent of surgical resection depends on tumor size, for small, superficial tumors (less than 5 mm), a simple wedge excision with primary closure may sufficient. Larger lesions may however require wide resections with lip reconstruction, necessitating the use of flaps [7]. Consequently cosmetic issues may result and more importantly disruption of the oral sphincter (orbicularis oris muscle), may lead to oral incompetence and in some cases to microstomia.

Radiation therapy, utilizing external beam and/or brachytherapy techniques, is an excellent alternative treatment option. In terms of disease control, results are similar to those of surgery [8]. However, in contrast to resection, functional and cosmetic outcomes are outstanding, and there are no significant additional toxicities [8-10].

Brachytherapy is a veteran technique and has been in clinical use for the past 100 years. The major advantage of brachytherapy is delivery of a high localized dose to the tumor, with a rapid dose fall-off in the adjacent normal tissues. In this manner, a high dose is delivered to the target with relative sparing of the surrounding healthy tissues. Another important advantage of brachytherapy is its short overall treatment time [8].

Various types of applicators and placement techniques have been used, including hypodermic needles, guide needles [11], plastic tubes, and guide gutters. Most commonly, the rigid or guide needle technique is used for lower lip cancers [11,12]. Since no randomized trials have been performed comparing the different modalities, it is not known if one technique is superior to the other. Most of the published experience to date comes from Low Dose Rate (LDR) brachytherapy. An overview of the literature shows for LDR Iridium-192 brachytherapy local control rates of 90–95% at 5 years [10]. Limited published data exists regarding High Dose Rate (HDR) treatment for lip cancer, However when comparing low dose rate (LDR) with HDR the long-term results were equally effective in local control and disease-free survival, but results with fewer complications when using HDR [11]. The scarcity of large trials with long-term results makes it difficult to determine the optimal dose and fractionation schedule.

Two trails of HDR brachytherapy for lip cancer appeared on a total of 67 patients, reporting 88-96% local control in 5 years, with excellent functional and cosmetic results [11,13]. Guinot et al. treated 99 patients with lip carcinoma with LDR and 104 with HDR brachytherapy. Local control at median follow-up above 5 years in both techniques revealed local contol of 94.9% and 95.2% respectively [11].

Finestres et al. treated 28 patients with HDR brachytherapy applied by superficial ready-made molds, without invasive needles. They reported 95% control at 46 month follow up [13]. Both studies present results similar to those obtained with LDR brachytherapy or surgery.

In 2009, The Head and Neck Working Group of the European Brachytherapy Group (GEC-ESTRO) published updated consensus recommendations. These recommendations are based mainly on the experience and publications of its members. In these guidelines, the rigid needle technique with a template is recommended for HDR brachytherapy, as it offers the best geometric conditions for the implant [8].

A recent report published in the Journal of Radiation Oncology describes the outcome of 51 patients treated with radioactive gold grain implantation for SCC of the lip. Most patients included in the trial had T1 lesions. Results at two-years of follow-up were excellent, both in terms of local recurrence and cosmetics [14].

The purpose of this work is to describe a novel technique used at our institution for brachytherapy of lip cancer and to illustrate its dosimetric advantage over the “classical” interstitial technique and the functional and cosmetic results.

## Methods and materials

### Patients

The study is a retrospective review of records of patients treated between 2005–2010 at the Hadassah University Hospital, Jerusalem, Israel. The study was approved by Institutional Ethic Committee (number 0125-11-HMO valid up to 4^th^ of June 2015). A Written informed consent was obtained from the patient whose pictures are presented in this publication.

Seven patients with a confirmed pathological diagnosis of squamous cell carcinoma, who were treated for lip cancer, using either a combination of external beam radiation therapy and HDR brachytherapy, or HDR brachytherapy alone, were included.

### The brachytherapy technique

**Figure 1:**
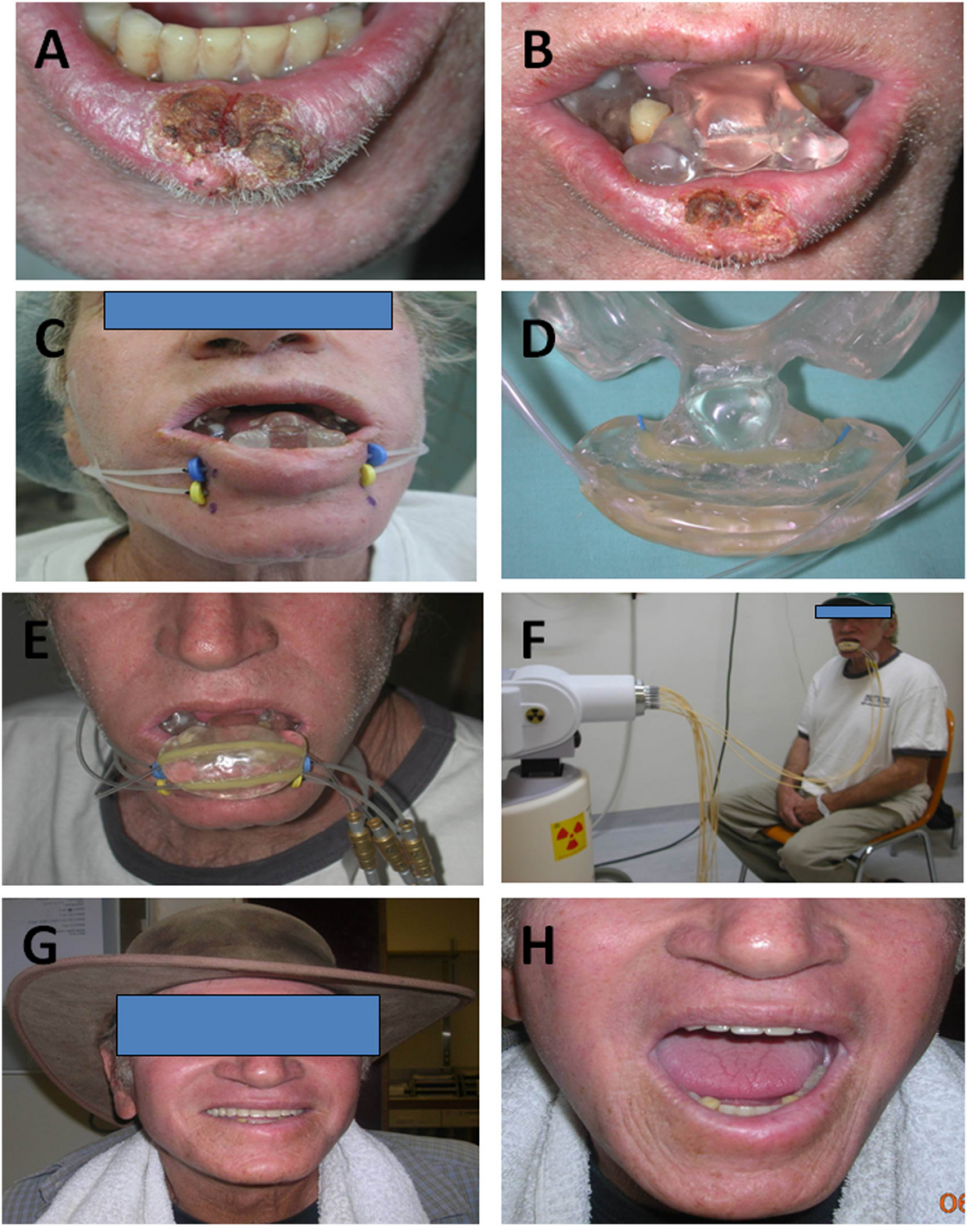
**Patient with lower lip T2 SCC during treatment: (A) pre treatment lesion, (B) mold used to distance the lip for external beam treatment, (C) the interstitial insertions, (D) CMS with embedded catheters, (E + F) the patient during treatment, (G + H) cosmetic and functional results after 4 years.** SCC = Squamous Cell Carcinoma CMS = Customized Mold Sandwich.

A technique using a combination of interstitial sleeve catheters and a surface mold with embedded sleeve catheters was employed. One to four flexible plastic interstitial catheters were implanted to the lip region, in a single-plane, with 1cm between the catheters. A customized acrylic mold was built to separate the lip from the adjacent bone (mandible or maxilla for lower or upper lip, respectively). When External Beam Radiation Therapy (EBRT) was given, this mold served to gain better normal tissue protection. The mold was used as a base for a saddle-shaped extension with embedded catheters and was placed over the patient’s lip (Figure 1).

**Figure 2:**
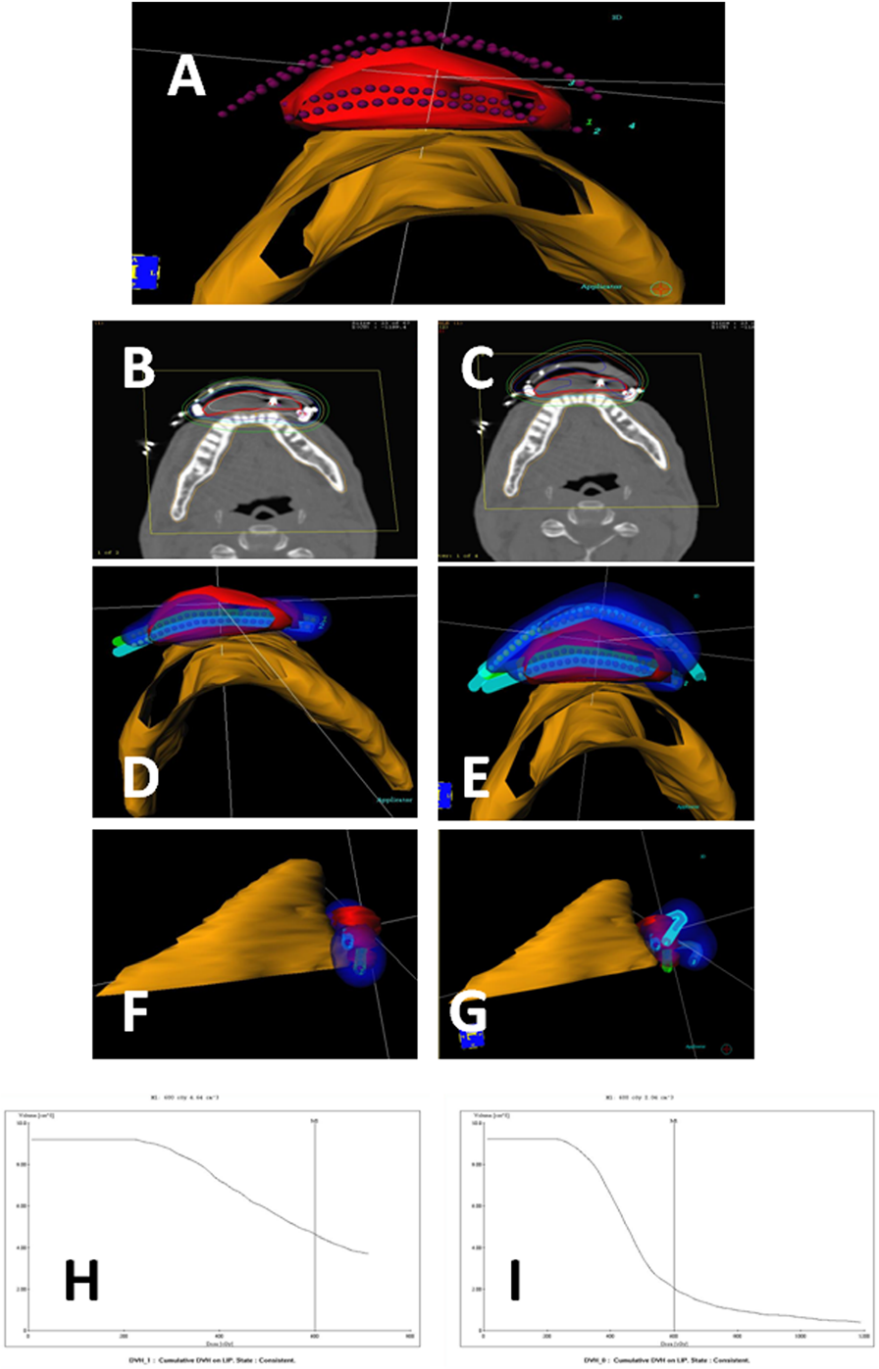
**Comparison between the "sandwich" technique and "classic" interstitial only technique.** Reconstructive image of the interstitial and mold catheters in relation to the lip and the mandible **(A)**. Comparison of the DVH between the interstitial catheters only **(B)** and the CMS **(C)** techniques at the same level. Note the invagination of the dose to the mandible in the “classic” interstitial catheters technique. Coverage by the lip catheters **(D,F)** compared to the CMS **(E,G)** from two different angles. The DVH of the target in the interstitial catheters only **(H)** and the CMS **(I)** techniques. Note that the high dose regions without the mold are more than twice that as with the mold. DVH = Dose Volume Histogram CMS = Customized Mold Sandwich.

Brachytherapy was delivered by 192- Iridium based Nucletron HDR afterloader, (Elekta AB, Stockholm, Sweden). CT simulation was used for 3D forward planning by Plato Brachytherapy planning system version 14.3.5, (Elekta AB, Stockholm, Sweden) [15]. The dose calculation algorithm of the PLATO planning system is based on the recommendations of AAPM task group 43 [16]. Target volumes and critical organs, including the mandible and lip were defined, and the interstitial catheters as well as the mold catheters were delineated (Figure 2). The treatment was given in a 2.5-3 Gy fractions BID, to a total dose of 25–42 Gy.

For evaluation of the catheter-baring mold contribution, we recalculated the dose with or without the mold, and compared the DVH of the plans, normalized to 3 Gy fraction.

### External Beam Radiation Therapy (EBRT)

Patients who had positive cervical lymph nodes or were at high risk for lymph node metastases, were initially treated with EBRT. CT simulation was used for treatment planning- ((Marconi Medical system M8000, Eclipse planning system (Varian, Palo Alto, California)). Intensity Modulated Radiation Therapy (IMRT) planning utilizing Analytical Anisotropic Algorithm (AAA) was used for treating the neck.

### Treatment evaluation

To estimate the CMS technique advantages over the commonly used “classical” interstitial technique, calculation was done to both techniques –in the same patient. The dose per fraction was normalized to 3 Gy. The DVH of the Clinical Tumor Volume (CTV), as well as dose to the mandible, were calculated. Dose Volume Histograms for the target volume and the surrounding normal tissue were generated. To evaluate the contribution of the catheter baring mold, we performed calculations using the interstitial sleeves only, ignoring the contribution of the sleeves embedded in the mold. The dose was reported according to the ICRU 58 guidelines [17], and the conformal index (COIN) [18] was calculated for each patient in both plans.

### Statistical methods

For statistics we used the Paired-Samples T test.

P < 0.05 was considered statistically significant.

## Results

### Patients

We identified seven patients who were treated with lip brachytherapy during the study period. All patients were male; the average age was 58 years (range 36–81). According to TNM staging, all patients had T2 lesions, ranging from 2 to 3 cm in size. All patients suffered from squamous cell carcinoma of the lip, six of them in the lower lip and one in the upper lip. Most of the patients had a well-differentiated histology, one patient had moderately to well-differentiated carcinoma, and one patient had a poorly-differentiated cancer.

All patients received radiation therapy as definitive treatment; one patient underwent surgical resection initially, and received brachytherapy alone for recurrent disease. Patient characteristics are summarized in Table 1.

**Table 1:** **Patient characteristics**

**Characteristic**	**Number (%)**
Age (years)	
Mean	58
Range	36-81
Sex	
Male	7 (100%)
Female	0 (0%)
Tumor site	
Upper lip	1 (14%)
Lower lip	6 (86%)
T stage	
T2	7 (100%)
Tumor size (cm)	
Mean	
Range	2-3
Tumor grade	
Well differentiated	5
Moderately differentiated	1
Ulcerated	1
Prior wedge resection	
(recurrence)	1
cm = centimeters	

Patients with positive or clinically suspicious neck nodes received external beam radiation to the neck as well. The median follow-up was 47 months (range: 41–59 months). Cosmetic and functional outcomes were extracted from doctor follow-up notes.

### The Customized Mold Sandwich (CMS) technique comparing to “classical” interstitial technique

Calculations based on ICRU 58 guidelines [17] showed treated Volume was in average 8.77cc for the CMS technique and 8.55cc for the “classical” interstitial only plan. The coverage of the CTV was 93% vs. 90% for the CMS over the “classical” interstitial technique respectively (P < 0.0004). The treated volume is the tissue volume that, based on the actual implant, receives at least 3Gy per fraction. The high-dose region is the volume encompass by isodose corresponding to 150% of the prescribed dose (V_150_). We found a statistically significant improvement of 20% (range 1-47%) in the high dose region favoring the CMS technique (p = 0.048). A low-dose region receiving 90% or less of the prescribed dose improved by 73% in favor of the CMS technique, however this was not statistically significant. A detailed individual dosimetry is shown in Table 2.

**Table 2:** **The plan characteristic reported according to ICRU 58**

		**“sandwich” technique**		**“Classic” interstitial**	
		**Volume (cc)**	**%**	**Volume (cc)**	**%**
Patient 1	Treated Volume	7.39	95.48%	7.29	94.19%
CTV-7.74cc	High-dose region	4.53	58.53%	5.91	76.36%
	Low-dose region	0.08	1.03%	0.25	3.23%
Patient 2	Treated Volume	6.49	92.71%	6.37	91.00%
CTV-7.00cc	High-dose region	3.7	52.86%	4.17	59.57%
	Low-dose region	0.33	4.71%	0.29	4.14%
Patient 3	Treated Volume	2.43	92.40%	2.35	89.35%
CTV-2.63cc	High-dose region	1.55	58.94%	1.57	59.70%
	Low-dose region	0.07	2.66%	0.11	4.18%
Patient 4	Treated Volume	8.75	97.01%	8.56	94.90%
CTV-9.02cc	High-dose region	4.92	54.55%	5.6	62.08%
	Low-dose region	0.11	1.22%	0.2	2.22%
Patient 5	Treated Volume	9.88	88.53%	9.63	86.29%
CTV-11.16cc	High-dose region	5.92	53.05%	7.01	62.81%
	Low-dose region	0.78	6.99%	1.15	10.30%
Patient 6	Treated Volume	8.64	93.81%	8.28	89.90%
CTV-9.21cc	High-dose region	4.96	53.85%	5.79	62.87%
	Low-dose region	0.2	2.17%	0.48	5.21%
Patient 7	Treated Volume	17.81	89.01%	17.36	86.76%
CTV-20.01cc	High-dose region	6.31	31.53%	9.3	46.48%
	Low-dose region	1.41	7.05%	1.19	5.95%

Calculation of the coverage of the target using the ICRU 58 guidelines for the “sandwich” technique and interstitial catheter only ("classic") for each individual patient.

When we calculated the dose to the maxilla or the mandible, the dose was negligible in all calculations due to the physical characteristics of the mold in distancing the lip. The Conformal Index (COIN) was calculated for all patients in both techniques and is shown in Table 3. An improvement of the index from an average of 0.88 to average of 0.92 (p = 0.043) was achieved by using the CMS technique.

**Table 3:** **The Conformal Index (COIN)**

	**“sandwich” technique**	**Interstitial catheter only**
Patient 1	0.930	0.784
Patient 2	0.927	0.910
Patient 3	0.924	0.894
Patient 4	0.970	0.949
Patient 5	0.885	0.863
Patient 6	0.938	0.887
Patient 7	0.884	0.868

Calculation of the Conformal index (COIN) for the “sandwich” technique and interstitial catheter only for each individual patient.

### Treatment toxicity

All patients developed a transitory mucositis and lip edema, which resolved within one month after the end of treatment. One patient developed a lip infection shortly after the end of treatment, and was treated with a one week course of antibiotics, and had an uneventful recovery.

Long term side effects were minimal and were limited to minimal fibrosis and dry lips. Skin defects were more likely to be the result of tumor resolution than radiation induced. No chronic ulceration or mandibular osteonecrosis were noted.

Excellent esthetic and functional results were obtained in all patients, including a smooth lip contour, effortless mouth opening, and clear speech. These outcome evaluations are based on both follow-up notes and patient judgment. See Table 4.

**Table 4:** **Dose, technique and outcomes**

**Patient**	**Tumor site**	**T stage**	**Previous treatments**	**DoseEBRT to lip (not inc. neck dose)**		**Brachytherapy dose**		**Implant Geometry**		**Follow up**	**`Cosmetic results 0-5 (5=excellent)**	**Functional results**
								**# interstitial sleeves**	**# external sleeves (MOLD)**			
1.	Lower lip	T2	none	50 Gy	2 Gy/fr	30 Gy	2 Gy BID	2	2	47 mo	5	5
2.	Upper lip	T2	none	50 Gy	2 Gy/fr	30 Gy	3 Gy BID	3	4	37 mo	5	5
3.	Lower lip	T2	none	none		42 Gy	3 Gy BID	1	2	32 mo	5	5
4.	Lower lip	T2	none	50 Gy	2 Gy/fr	18 Gy	3 Gy BID	2	3	33 mo	5	5
5.	Lower lip	T2	Surgery, close margins	44 Gy	2 Gy/fr	25 Gy	2.5 Gy BID	2	2	29 mo	5	5
6.	Lower lip	T2	none	none		42 Gy	3 Gy BID	4	3	38 mo	5	5
7.	Lower lip recurrence	T2 post op	Wedge resection	none		39 Gy	3 Gy BID	2	1	29 mo	5	5

EBRT = External Beam Radiation Therapy, Gy = Gray, fr = fraction, BID = Twice daily, mo = month, #=number of.

## Discussion

The CMS technique, which combines interstitial catheters with a saddle-shaped mold with embedded catheters, result, in better tumor coverage and conformality, compared to the “classical” interstitial technique. The addition of the mold enabled relative sparing of the mandible/maxilla and adjacent normal tissues by reserving the region of treatment (i.e. lip) from the bone. Since we disregard the catheters in the mold during the calculation of the “classical” solution, but not the mold itself with its advantages, additional benefit was not reflected in the comparison of the mandible dose calculations. Our technique resulted in excellent tumor control rates with minimal short and long-term toxicity.

Finestres et al. [13] reported on their experience with 28 patients treated with HDR brachytherapy by means of acrylic applicators without interstitial catheter insertions. All patients had complete remission of their tumor; excellent cosmetic results were reported, with no late complications. Guinot et al. [11] reported treatment for patient with T1-T4 lesions using parallel needles only, with a template forming a triangular distribution. They reported a local control rate of 90%. In our study, we report a technique that combines the advantages of both methods, a combination of interstitial catheters and a surface mold. This enabled improving the CTV coverage and dose homogeneity, without the need to increase the number of interstitial sleeves. This may contribute to the excellent long term outcome in terms of tumor control cosmetics and functionality.

Weak points in our study include it being retrospective, and consequently the need to use the same planning CT for comparison calculations. Therefore, the lip is pushed away from the mandible by the mold, not only for the calculation of the CMS technique, but also for the calculation of the “classic” technique, thus underscore the advantage that the mold achieves.

## Conclusion

Compared to the “classical” interstitial only technique, the “sandwich” technique of interstitial brachytherapy with a dedicated mold, achieves better dose volume distribution, better treatment homogenously and better normal tissue protection.
